# Supplementary material for: Impact of postoperative macular comorbidity on visual outcomes after Descemet’s membrane endothelial keratoplasty: A multicenter analysis
Source: PLoS One. 2026 Feb 26;21(2):e0343883. doi: 10.1371/journal.pone.0343883 (PMC12944715; doi:10.1371/journal.pone.0343883)
Supplement: S1 Table — (PDF) [file pone.0343883.s001.pdf]

Case number	Patient numb	right_left	age_at_surge	sex	disease	surgery_type	rejection_eve	rebubbling	tripple	lens_status	preoperative_	preoperative_	final_ECD	days_follow_	visual_acuity_	visual_acuity_	cme	erm	wet_amd	macular_retin	group
1	1	[2] Left	84	[2] Female	[1] FECD	[1] DMEK	[1] No	[1] No	[1] DMEK	[1] IOL	0.3	0.522878745	588	546	0.6	0.22184875	[1] No	[2] Yes	[1] No	[2] Yes	[1] FECD
2	2	[1] Right	81	[2] Female	[1] FECD	[1] DMEK	[1] No	[1] No	[1] DMEK	[1] IOL	0.8	0.096910013	676	2045	1.2	-0.07918125	[1] No	[2] Yes	[1] No	[2] Yes	[1] FECD
3	3	[2] Left	74	[2] Female	[2] BK	[2] DMEK / DN	[1] No	[1] No	[1] DMEK	[1] IOL	0.01	2	947	260	0.9	0.045757491	[2] Yes	[2] Yes	[1] No	[2] Yes	[2] BK
4	4	[2] Left	83	[2] Female	[2] BK	[1] DMEK	[1] No	[1] No	[1] DMEK	[2] Scleral fixation	0.01	2	805	1684	0.15	0.823908741	[1] No	[1] No	[2] Yes	[2] Yes	[2] BK
5	5	[2] Left	65	[2] Female	[2] BK	[1] DMEK	[1] No	[1] No	[1] DMEK	[1] IOL	0.1	1	720	1579	0.9	0.045757491	[1] No	[1] No	[1] No	[1] No	[2] BK
6	6	[2] Left	56	[2] Female	[2] BK	[1] DMEK	[1] No	[1] No	[1] DMEK	[2] Scleral fixation	0.3	0.522878745	722	2711	0.6	0.22184875	[2] Yes	[1] No	[1] No	[2] Yes	[2] BK
7	7	[2] Left	74	[1] Male	[2] BK	[1] DMEK	[1] No	[1] No	[1] DMEK	[1] IOL	0.3	0.522878745	1730	2056	0.6	0.22184875	[1] No	[2] Yes	[1] No	[2] Yes	[2] BK
8	8	[1] Right	57	[1] Male	[2] BK	[1] DMEK	[1] No	[2] Yes	[1] DMEK	[1] IOL	0.3	0.522878745	1055	2549	0.7	0.15490196	[2] Yes	[1] No	[1] No	[2] Yes	[2] BK
9	9	[1] Right	82	[2] Female	[2] BK	[1] DMEK	[1] No	[1] No	[1] DMEK	[1] IOL	0.05	1.301029996	877	2231	1	0	[1] No	[1] No	[1] No	[1] No	[2] BK
10	9	[2] Left	82	[2] Female	[2] BK	[1] DMEK	[2] Yes	[1] No	[1] DMEK	[1] IOL	0.03	1.522878745	601	2290	0.8	0.096910013	[1] No	[1] No	[1] No	[1] No	[2] BK
11	10	[2] Left	86	[2] Female	[2] BK	[1] DMEK	[1] No	[1] No	[1] DMEK	[1] IOL	0.3	0.522878745	937	337	0.5	0.301029996	[1] No	[2] Yes	[1] No	[2] Yes	[2] BK
12	10	[1] Right	81	[2] Female	[2] BK	[1] DMEK	[1] No	[1] No	[1] DMEK	[1] IOL	0.01	2	940	2395	0.7	0.15490196	[1] No	[2] Yes	[1] No	[2] Yes	[2] BK
13	11	[2] Left	69	[2] Female	[1] FECD	[1] DMEK	[1] No	[2] Yes	[1] DMEK	[1] IOL	0.3	0.522878745	787	2293	0.7	0.15490196	[1] No	[1] No	[1] No	[1] No	[1] FECD
14	12	[1] Right	74	[2] Female	[1] FECD	[1] DMEK	[1] No	[1] No	[1] DMEK	[1] IOL	0.05	1.301029996	609	2223	1	0	[1] No	[1] No	[1] No	[1] No	[1] FECD
15	13	[2] Left	76	[2] Female	[2] BK	[1] DMEK	[1] No	[1] No	[1] DMEK	[1] IOL	0.3	0.522878745	639	1643	0.5	0.301029996	[2] Yes	[1] No	[1] No	[2] Yes	[2] BK
16	13	[1] Right	75	[2] Female	[2] BK	[1] DMEK	[1] No	[1] No	[1] DMEK	[1] IOL	0.4	0.397940009	792	1993	0.7	0.15490196	[2] Yes	[1] No	[1] No	[2] Yes	[2] BK
17	14	[2] Left	73	[2] Female	[1] FECD	[1] DMEK	[1] No	[2] Yes	[1] DMEK	[1] IOL	0.3	0.522878745	1785	510	0.7	0.15490196	[1] No	[1] No	[1] No	[1] No	[1] FECD
18	14	[1] Right	73	[2] Female	[1] FECD	[1] DMEK	[1] No	[2] Yes	[1] DMEK	[1] IOL	0.04	1.397940009	1934	660	0.8	0.096910013	[1] No	[1] No	[1] No	[1] No	[1] FECD
19	15	[2] Left	73	[2] Female	[1] FECD	[1] DMEK	[1] No	[2] Yes	[1] DMEK	[1] IOL	0.3	0.522878745	622	607	0.8	0.096910013	[1] No	[1] No	[1] No	[1] No	[1] FECD
20	16	[1] Right	81	[2] Female	[2] BK	[1] DMEK	[1] No	[1] No	[1] DMEK	[1] IOL	0.1	1	360	1922	0.4	0.397940009	[1] No	[1] No	[1] No	[1] No	[2] BK
21	17	[1] Right	71	[1] Male	[1] FECD	[1] DMEK	[1] No	[2] Yes	[1] DMEK	[1] IOL	0.2	0.698970004	465	1458	0.5	0.301029996	[2] Yes	[1] No	[1] No	[2] Yes	[1] FECD
22	17	[2] Left	71	[1] Male	[1] FECD	[2] DMEK / DN	[1] No	[1] No	[1] DMEK	[1] IOL	0.2	0.698970004	1099	473	0.4	0.397940009	[2] Yes	[2] Yes	[1] No	[2] Yes	[1] FECD
23	18	[2] Left	84	[2] Female	[1] FECD	[1] DMEK	[1] No	[2] Yes	[1] DMEK	[1] IOL	0.06	1.22184875	722	1100	0.9	0.045757491	[1] No	[1] No	[1] No	[1] No	[1] FECD
24	18	[1] Right	83	[2] Female	[1] FECD	[1] DMEK	[1] No	[1] No	[1] DMEK	[1] IOL	0.5	0.301029996	1785	1156	0.9	0.045757491	[1] No	[1] No	[1] No	[1] No	[1] FECD
25	19	[2] Left	76	[2] Female	[2] BK	[1] DMEK	[1] No	[1] No	[1] DMEK	[1] IOL	0.2	0.698970004	682	1551	0.4	0.397940009	[1] No	[2] Yes	[1] No	[2] Yes	[2] BK
26	20	[1] Right	75	[2] Female	[2] BK	[1] DMEK	[1] No	[1] No	[1] DMEK	[1] IOL	0.1	1	783	855	0.6	0.22184875	[1] No	[1] No	[2] Yes	[2] Yes	[2] BK
27	21	[2] Left	76	[2] Female	[1] FECD	[1] DMEK	[2] Yes	[1] No	[1] DMEK	[1] IOL	0.1	1	760	2584	0.4	0.397940009	[1] No	[1] No	[1] No	[1] No	[1] FECD
28	22	[2] Left	87	[2] Female	[1] FECD	[1] DMEK	[1] No	[1] No	[1] DMEK	[1] IOL	0.1	1	1336	231	0.5	0.301029996	[1] No	[1] No	[1] No	[1] No	[1] FECD
29	22	[1] Right	87	[2] Female	[1] FECD	[1] DMEK	[1] No	[1] No	[1] DMEK	[1] IOL	0.2	0.698970004	726	399	0.4	0.397940009	[1] No	[1] No	[1] No	[1] No	[1] FECD
30	23	[2] Left	55	[2] Female	[1] FECD	[1] DMEK	[1] No	[1] No	[1] DMEK	[1] IOL	0.9	0.045757491	786	620	1	0	[1] No	[2] Yes	[1] No	[2] Yes	[1] FECD
31	24	[2] Left	72	[1] Male	[2] BK	[1] DMEK	[1] No	[1] No	[1] DMEK	[1] IOL	0.01	2	452	683	0.03	1.522878745	[2] Yes	[1] No	[1] No	[2] Yes	[2] BK
32	25	[1] Right	78	[2] Female	[2] BK	[1] DMEK	[1] No	[1] No	[1] DMEK	[1] IOL	0.01	2	542	715	0.2	0.698970004	[2] Yes	[2] Yes	[1] No	[2] Yes	[2] BK
33	26	[2] Left	85	[2] Female	[1] FECD	[1] DMEK	[1] No	[2] Yes	[1] DMEK	[1] IOL	0.3	0.522878745	617	438	0.5	0.301029996	[1] No	[1] No	[1] No	[1] No	[1] FECD
34	26	[1] Right	73	[2] Female	[1] FECD	[1] DMEK	[1] No	[2] Yes	[1] DMEK	[1] IOL	0.3	0.522878745	746	1069	0.7	0.15490196	[2] Yes	[1] No	[1] No	[2] Yes	[1] FECD
35	27	[1] Right	83	[2] Female	[1] FECD	[1] DMEK	[1] No	[2] Yes	[1] DMEK	[1] IOL	0.2	0.698970004	631	687	0.8	0.096910013	[1] No	[1] No	[1] No	[1] No	[1] FECD
36	28	[2] Left	61	[2] Female	[1] FECD	[1] DMEK	[1] No	[1] No	[1] DMEK	[1] IOL	0.2	0.698970004	622	757	0.9	0.045757491	[1] No	[1] No	[1] No	[1] No	[1] FECD
37	28	[1] Right	60	[2] Female	[1] FECD	[1] DMEK	[1] No	[1] No	[1] DMEK	[1] IOL	0.4	0.397940009	629	932	1	0	[1] No	[1] No	[1] No	[1] No	[1] FECD
38	29	[2] Left	78	[2] Female	[1] FECD	[4] DMEK / DS	[1] No	[1] No	[1] DMEK	[1] IOL	0.02	1.698970004	746	1024	0.2	0.698970004	[1] No	[2] Yes	[1] No	[2] Yes	[1] FECD
39	30	[1] Right	62	[1] Male	[1] FECD	[2] DMEK / DN	[1] No	[1] No	[1] DMEK	[1] IOL	0.3	0.522878745	621	4143	1.2	-0.07918125	[1] No	[2] Yes	[1] No	[2] Yes	[1] FECD
40	31	[1] Right	68	[2] Female	[2] BK	[1] DMEK	[1] No	[1] No	[1] DMEK	[1] IOL	0.1	1	861	1495	1.2	-0.07918125	[2] Yes	[1] No	[1] No	[2] Yes	[2] BK
41	32	[2] Left	69	[1] Male	[2] BK	[1] DMEK	[1] No	[1] No	[1] DMEK	[2] Scleral fixation	0.6	0.22184875	675	2360	1.2	-0.07918125	[1] No	[2] Yes	[1] No	[2] Yes	[2] BK
42	33	[1] Right	73	[1] Male	[1] FECD	[1] DMEK	[1] No	[1] No	[1] DMEK	[1] IOL	0.3	0.522878745	1049	357	1	0	[1] No	[1] No	[1] No	[1] No	[1] FECD
43	34	[2] Left	74	[2] Female	[2] BK	[1] DMEK	[1] No	[1] No	[1] DMEK	[1] IOL	0.3	0.522878745	853	385	1.2	-0.07918125	[1] No	[1] No	[1] No	[1] No	[2] BK
44	35	[1] Right	58	[2] Female	[2] BK	[1] DMEK	[1] No	[1] No	[1] DMEK	[1] IOL	0.1	1	1501	232	0.8	0.096910013	[1] No	[1] No	[1] No	[1] No	[2] BK
45	36	[1] Right	81	[2] Female	[1] FECD	[1] DMEK	[1] No	[1] No	[1] DMEK	[1] IOL	0.1	1	855	231	0.5	0.301029996	[1] No	[1] No	[1] No	[1] No	[1] FECD
46	37	[1] Right	71	[1] Male	[1] FECD	[1] DMEK	[1] No	[2] Yes	[1] DMEK	[1] IOL	0.3	0.522878745	1898	217	0.6	0.22184875	[1] No	[2] Yes	[1] No	[2] Yes	[1] FECD
47	38	[2] Left	85	[2] Female	[2] BK	[1] DMEK	[1] No	[2] Yes	[1] DMEK	[1] IOL	0.05	1.301029996	1292	98	0.8	0.096910013	[1] No	[2] Yes	[1] No	[2] Yes	[2] BK
48	39	[2] Left	68	[2] Female	[1] FECD	[1] DMEK	[1] No	[1] No	[1] DMEK	[1] IOL	0.3	0.522878745	1345	212	0.6	0.22184875	[1] No	[2] Yes	[1] No	[2] Yes	[1] FECD
49	40	[2] Left	78	[2] Female	[1] FECD	[1] DMEK	[1] No	[1] No	[1] DMEK	[1] IOL	0.2	0.698970004	791	619	0.9	0.045757491	[1] No	[1] No	[1] No	[1] No	[1] FECD
50	41	[1] Right	74	[1] Male	[2] BK	[1] DMEK	[1] No	[1] No	[1] DMEK	[1] IOL	0.9	0.045757491	683	871	1	0	[1] No	[1] No	[1] No	[1] No	[2] BK
51	42	[1] Right	72	[2] Female	[1] FECD	[2] DMEK / DN	[1] No	[1] No	[1] DMEK	[1] IOL	0.4	0.397940009	666	1200	1.2	-0.07918125	[1] No	[1] No	[1] No	[1] No	[1] FECD
52	43	[2] Left	58	[2] Female	[2] BK	[2] DMEK / DN	[1] No	[1] No	[1] DMEK	[1] IOL	0.2	0.698970004	1203	192	0.5	0.301029996	[1] No	[2] Yes	[1] No	[2] Yes	[2] BK
53	44	[1] Right	56	[1] Male	[1] FECD	[1] DMEK	[1] No	[1] No	[1] DMEK	[1] IOL	0.9	0.045757491	524	1998	1.2	-0.07918125	[1] No	[1] No	[1] No	[1] No	[1] FECD
54	44	[2] Left	56	[1] Male	[1] FECD	[1] DMEK	[1] No	[1] No	[1] DMEK	[1] IOL	0.3	0.522878745	1625	2152	1.2	-0.07918125	[1] No	[1] No	[1] No	[1] No	[1] FECD
55	45	[1] Right	79	[2] Female	[2] BK	[1] DMEK	[1] No	[1] No	[1] DMEK	[1] IOL	0.15	0.823908741		241	0.5	0.301029996	[2] Yes	[1] No	[1] No	[2] Yes	[2] BK
56	46	[1] Right	70	[2] Female	[2] BK	[1] DMEK	[1] No	[1] No	[1] DMEK	[1] IOL	0.8	0.096910013	789	2055	0.7	0.15490196	[1] No	[1] No	[1] No	[1] No	[2] BK
57	47	[1] Right	81	[1] Male	[2] BK	[1] DMEK	[1] No	[1] No	[1] DMEK	[1] IOL	0.2	0.698970004	1102	1314	0.3	0.522878745	[1] No	[1] No	[1] No	[1] No	[2] BK
58	48	[1] Right	70	[2] Female	[2] BK	[1] DMEK	[1] No	[1] No	[1] DMEK	[1] IOL	0.6	0.22184875	1607	507	0.7	0.15490196	[1] No	[1] No	[1] No	[1] No	[2] BK
59	49	[2] Left	64	[1] Male	[1] FECD	[1] DMEK	[1] No	[1] No	[1] DMEK	[1] IOL	0.3	0.522878745	621	4029	0.9	0.045757491	[1] No	[1] No	[1] No	[1] No	[1] FECD
60	50	[2] Left	52	[1] Male	[1] FECD	[1] DMEK	[1] No	[1] No	[1] DMEK	[1] IOL	0.3	0.522878745	1629	2805	1	0	[1] No	[1] No	[1] No	[1] No	[1] FECD
61	51	[1] Right	48	[1] Male	[1] FECD	[1] DMEK	[1] No	[2] Yes	[1] DMEK	[1] IOL	0.2	0.698970004	695	4227	1	0	[1] No	[1] No	[1] No	[1] No	[1] FECD
62	52	[1] Right	57	[2] Female	[1] FECD	[1] DMEK	[1] No	[1] No	[1] DMEK	[1] IOL	0.5	0.301029996	784	3415	1.2	-0.07918125	[1] No	[1] No	[2] Yes	[2] Yes	[1] FECD
63	53	[2] Left	61	[1] Male	[2] BK	[1] DMEK	[1] No	[1] No	[1] DMEK	[1] IOL	0.5	0.301029996	518	2504	0.7	0.15490196	[1] No	[1] No	[1] No	[1] No	[2] BK
64	54	[1] Right	60	[1] Male	[2] BK	[1] DMEK	[1] No	[1] No	[1] DMEK	[1] IOL	0.7	0.15490196	467	2938	0.9	0.045757491	[1] No	[1] No	[1] No	[1] No	[2] BK
65	55	[1] Right	70	[2] Female	[2] BK	[1] DMEK	[1] No	[1] No	[1] DMEK	[1] IOL	0.4	0.397940009	771	1914	0.9	0.045757491	[1] No	[1] No	[1] No	[1] No	[2] BK
66	56	[2] Left	74	[1] Male	[2] BK	[1] DMEK	[1] No	[2] Yes	[1] DMEK	[1] IOL	0.5	0.301029996	1580	1914	1	0	[1] No	[1] No	[1] No	[1] No	[2] BK
67	57	[1] Right	79	[2] Female	[2] BK	[1] DMEK	[1] No	[1] No	[1] DMEK	[1] IOL	0.7	0.15490196	704	1781	0.8	0.096910013	[1] No	[1] No	[1] No	[1] No	[2] BK
68	58	[1] Right	75	[1] Male	[1] FECD	[1] DMEK	[1] No	[1] No	[1] DMEK	[1] IOL	0.6	0.22184875	1443	914	1	0	[1] No	[2] Yes	[1] No	[2] Yes	[1] FECD
69	59	[1] Right	61	[1] Male	[2] BK	[1] DMEK	[1] No	[1] No	[1] DMEK	[1] IOL	0.3	0.522878745	554	2740	0.8	0.096910013	[1] No	[1] No	[1] No	[1] No	[2] BK
70	60	[1] Right	69	[1] Male	[2] BK	[1] DMEK	[1] No	[1] No	[1] DMEK	[1] IOL	0.3	0.522878745	1446	2735	1.2	-0.07918125	[1] No	[1] No	[1] No	[1] No	[2] BK
71	61	[1] Right	72	[2] Female	[2] BK	[1] DMEK	[1] No	[1] No	[1] DMEK	[1] IOL	0.4	0.397940009	668	2222	0.9	0.045757491	[1] No	[1] No	[2] Yes	[2] Yes	[2] BK
72	62	[2] Left	77	[2] Female	[2] BK	[1] DMEK	[1] No	[1] No	[1] DMEK	[1] IOL	0.3	0.522878745	1256	2110	0.7	0.15490196	[1] No	[2] Yes	[1] No	[2] Yes	[2] BK
73	63	[1] Right	75	[1] Male	[2] BK	[1] DMEK	[1] No	[1] No	[1] DMEK	[1] IOL	0.3	0.522878745	1083	1704	1.2	-0.07918125	[1] No	[1] No	[1] No	[1] No	[2] BK
74	63	[2] Left	74	[1] Male	[2] BK	[1] DMEK	[1] No	[1] No	[1] DMEK	[1] IOL	0.7	0.15490196	1371	1865	1	0	[1] No	[1] No	[1] No	[1] No	[2] BK
75	64	[1] Right	77	[2] Female	[2] BK	[1] DMEK	[1] No	[1] No	[1] DMEK	[1] IOL	0.7	0.15490196	974	563	1	0	[1] No	[1] No	[1] No	[1] No	[2] BK
76	65	[2] Left	79	[2] Female	[2] BK	[1] DMEK	[1] No	[1] No	[1] DMEK	[1] IOL	0.06	1.22184875	1153	871	1	0	[1] No	[1] No	[1] No	[1] No	[2] BK
77	66	[1] Right	76	[2] Female	[2] BK	[1] DMEK	[1] No	[1] No	[1] DMEK	[1] IOL	0.02	1.698970004	1010	1102	0.7	0.15490196	[1] No	[1] No	[1] No	[1] No	[2] BK
